# In Silico Model Estimates the Clinical Trial Outcome of Cancer Vaccines

**DOI:** 10.3390/cells10113048

**Published:** 2021-11-05

**Authors:** Orsolya Lőrincz, József Tóth, Levente Molnár, István Miklós, Kata Pántya, Mónika Megyesi, Eszter Somogyi, Zsolt Csiszovszki, Enikő R. Tőke

**Affiliations:** 1 Treos Bio Ltd., London W1W6XB, UK; orsolya.lorincz@treosbio.com (O.L.); jozsef.toth@treosbio.com (J.T.); levente.molnar@treosbio.com (L.M.); miklos.istvan.74@gmail.com (I.M.); kata.pantya@treosbio.com (K.P.); lakatosmonika@yahoo.com (M.M.); eszter.somogyi@treosbio.com (E.S.); zsolt.csiszovszki@treosbio.com (Z.C.); 2 Treos Bio Zrt, 8200 Veszprém, Hungary; 3 Alfréd Rényi Institute of Mathematics, Eötvös Loránd Research Network, 1053 Budapest, Hungary; 4 Computer Science and Automation Research Institute (SZTAKI), Eötvös Loránd Research Network, 1111 Budapest, Hungary

**Keywords:** cancer vaccine, HLA genotype, in silico trial, immune response rate, clinical response rate

## Abstract

Over 30 years after the first cancer vaccine clinical trial (CT), scientists still search the missing link between immunogenicity and clinical responses. A predictor able to estimate the outcome of cancer vaccine CTs would greatly benefit vaccine development. Published results of 94 CTs with 64 therapeutic vaccines were collected. We found that preselection of CT subjects based on a single matching HLA allele does not increase immune response rates (IRR) compared with non-preselected CTs (median 60% vs. 57%, *p* = 0.4490). A representative in silico model population (MP) comprising HLA-genotyped subjects was used to retrospectively calculate in silico IRRs of CTs based on the percentage of MP-subjects having epitope(s) predicted to bind ≥ 1–4 autologous HLA allele(s). We found that in vitro measured IRRs correlated with the frequency of predicted multiple autologous allele-binding epitopes (AUC 0.63–0.79). Subgroup analysis of multi-antigen targeting vaccine CTs revealed correlation between clinical response rates (CRRs) and predicted multi-epitope IRRs when HLA threshold was ≥ 3 (*r* = 0.7463, *p* = 0.0004) but not for single HLA allele-binding epitopes (*r* = 0.2865, *p* = 0.2491). Our results suggest that CRR depends on the induction of broad T-cell responses and both IRR and CRR can be predicted when epitopes binding to multiple autologous HLAs are considered.

## 1. Introduction

Based on the proposed mechanism of action of cancer vaccines, one could expect the T-cell mediated killing of malignant cells and thus shrinkage of the tumor. However, to date no clinical trials (CTs) have convincingly shown association between immune response rate (IRR) and clinical response rate (CRR) in terms of tumor shrinkage [1,2], rather survival benefit was found to correlate in some cases [3,4,5,6]. These results suggest that eliciting broad and robust immune responses in high proportion of subjects should still be the focus of cancer vaccine development.

The active substances of cancer vaccines are immunogenic epitopes of tumor-associated or tumor-specific proteins. An epitope is a short, 8–25 amino acid long peptide fragment derived from a protein, specifically bound to a human leukocyte antigen (HLA) molecule and consequently can induce immune responses against the diseased cells that express the same peptide. The direct involvement of HLA molecules (major histocompatibility complex, MHC) in T-cell recognition of antigens was first shown in 1974 by Zinkernagel and Doherty [7,8], who received the Nobel Prize for their pioneer work in 1996. In 1987 Wiley and co-workers provided the explanation, which brought forth a paradigm shift, not only in the HLA field but also in immunology in general [9,10]. Since then, an era of therapeutic vaccines started and today many researchers focus on designing vaccines based on HLA allele-binding predictions. HLAs are encoded by the most polymorphic genes of the human genome. Each person has a maternal and a paternal allele for the three HLA class I molecules (HLA-A*, HLA-B*, HLA-C*). Practically, each person expresses a different combination of six HLA class I molecules that present different epitopes from the same antigen (protein). The current challenge is the accurate prediction of epitopes that induce CD8^+^ cytotoxic T-cell (CTL) responses, using computational approaches. Peptides with a predicted HLA-binding affinity in the strong range (half-maximal inhibitory concentration (IC50) < 150 nmol/L) are considered more likely to induce CD8^+^ T-cell responses [11]. The recognition of HLA-presented epitopes by T-cell receptors (TCR) is also a determining part of efficient immune response generation. The heterogeneity of TCRs in a subject has major impact on the immune response; for instance, a single HLA class I influenza epitope was found to produce a few hundreds of different TCRs in each subject while a single HLA class II cancer epitope generated 8–16 different TCR clonotypes with different relative abundance in four cancer patients [12,13]. Another comprehensive study also demonstrated that TCRs specific to the same epitope can be more diverse than TCRs recognizing different epitopes [14]. The models for predicting recognition between TCRs and epitopes are continuously evolving and their reliability is varying [15]. The performance of computational epitope-HLA binding prediction tools is well characterized and high specificity and accuracy is reported for algorithms that are based on either position-specific scoring matrix, neural network, or consensus methods [16]. Therefore, the lack of correlation between HLA-binding predictions and immune responses may not be attributed to the insufficiency of predictors, rather to a missing link between the epitope-HLA binding and the activation of the epitope-specific T-cell within the mechanism of eliciting immune response. This is supported by the finding of a study that less than 1% of predicted strong-binder epitopes were recognized by T-cells [17]. Many therapeutic cancer vaccines are designed by selecting epitopes derived from tumor-specific proteins that bind to a specific HLA allele (e.g., A*02:01), or an allele group (e.g., A02) in order to be immunogenic in a broad population. This became a common practice since ~90% of the United States (US) population and ~85% of the world population are positive for at least one of the six most prevalent HLA types (A*02:01, A*01:01, A*03:01, A*11:01, A*24:02, and B*07:02) [18,19,20]. For many vaccines designed following such an approach the expression of the specific allele is also used as enrollment criteria for trial subjects [21,22,23,24]. However, the IRR obtained in such cancer vaccine CTs range from ~15% to 100% in unpredictable fashion.

Recent technological advances in predicting HLA-binding neoepitopes from mutation-derived tumor neoantigen have enabled development of more effective patient-specific therapeutic vaccines. However, also in these neoantigen vaccines, only 16–20% of the predicted neoepitopes induce CD8^+^ T-cell responses and the majority of peptides included in the personalized vaccines proved to be false positive [25,26,27,28]. Interestingly, the CD4^+^ T-cell responses were more remarkable for each vaccine. Compared with CD8^+^ killer T-cells, where the HLA-bound peptide serves as direct activation signal towards the CTL activation, CD4^+^ T-cells have multiple indirect roles in vaccine-induced immune responses by enhancing the differentiation of CD8^+^ effector T-cells and producing Th1 cytokines facilitating the antitumor responses by e.g., recruiting macrophages and natural killer (NK) cells. Growing evidence suggests that CD4^+^ T-cells also have a killing function, but this subset is not dominant [13]. However, HLA class II epitope prediction is less accurate compared to HLA class I epitope prediction, because of the highly variable epitope length (12–25 amino acids) and the enrichment of overlapping epitopes at the same protein region [29].

To overcome the limited immunogenicity of vaccine peptide selection, HLA-presented neoepitopes were predicted on the surface of the patient’s tumor cells [30]. However, from 20 predicted neoepitopes only two per patient induced CD8^+^ T-cells (in the responder subgroup). A bioassay screening the preexisting patient-relevant neoantigen T-cell responses in an HLA-agnostic way improved the true positive rate of selected peptides to 59% in terms of CD8^+^ T-cell responses [31]. However, neoepitope identification approaches are complex, time-consuming, and not feasible for each tumor and tumor-type [32]. Therefore, cancer vaccine development requires substantial improvement in prediction of epitopes that induce T-cell responses in individuals and consequently in larger populations (CTs) as well.

Here, we present an in silico model that is able to predict the clinical outcome of cancer vaccine CTs based on a novel immunological concept and a representative HLA-genotyped model population. This meta-analysis of almost a hundred CTs with therapeutic vaccines indicates that not only a single, but all six HLA class I alleles of individuals should be taken into consideration in relation to predicted antigen-specific immune responses.

## 2. Materials and Methods

### 2.1. Studies Included in the Meta-Analysis

The literature search was conducted between December 2016 to March 2019 in English language using PubMed and Google Scholar search engines. Peer-reviewed publications providing CD8^+^ T-cell immune response and/or clinical response data were eligible for the present study. Other inclusion criteria were as follows: the vaccine antigen sequence was disclosed or otherwise available. Studies cited by the eligible articles that contained data using the same vaccine were also searched and filtered for eligibility. Eligible therapeutic vaccine types were peptide (at least nine amino acids long), nucleic acid-based, or peptide-loaded dendritic cell vaccines. Protein vaccines (whole proteins or long peptides comprising >50 amino acids) were excluded since those have different mechanism of action.

Studies were included if they used any of the following immunoassays: interferon-gamma (IFN-γ) ELISPOT or enzyme-linked immunosorbent assay (ELISA), MHC multimer, T-cell proliferation, intracellular cytokine staining, or cytotoxicity (killing) assays with the following restrictions: (1) in case the test antigen used for the immune response measurement was whole protein or long peptide, the data were only eligible for the analysis if the CD8^+^ phenotype of responsive cells was proven (e.g.,: flow cytometry or CD4^+^ T-cell depletion), (2) if more immunoassays were used, the chosen method was the one which procedure contained the fewest in vitro stimulation rounds or which the investigator used for responder identification in the publication, (3) if more than one round of in vitro stimulation was performed, the results were excluded, as stimulating multiple times can heavily bias immunoassay results. For clinical response assessment, CTs using the following standards were eligible: Response Evaluation Criteria in Solid Tumors (RECIST), World Health Organization (WHO), International Working Group (IWG), or Cancer and Leukemia Group B (CALGB) criteria [33,34,35,36]. The following exclusion criteria were used: chemotherapy combinations were excluded if their mechanism of action affected CD8^+^ T-cell responses; and delayed-type hypersensitivity (DTH) assays for immunogenicity assessment. A review of abstracts identified 185 papers that were possibly relevant. Of these, 93 were excluded since they did not fulfil the pre-defined criteria described above. The remaining 92 papers, covering a total of 94 CTs were processed for further analysis and data extraction. The 94 CTs contained response data of 2338 subjects treated with 64 immunotherapeutic vaccines (63 cancer/neoplasia and one human immunodeficiency virus [HIV] vaccine), which targeted a total of 88 different antigens. Table 1 and Table A1 collect the selected CTs.

IRR is the proportion of subjects in the study population who had in vitro CD8^+^ T-cell responses induced by the study vaccine as reported in the publications. CRR is the proportion of subjects in the study population who had clinical response (partial or complete in terms of tumor shrinkage for solid tumors and reduction in M-component level or myeloblasts in the bone marrow for hematological tumors) after vaccination as reported in the publications (Table A1).

### 2.2. In Silico Trial

The in silico trial is based on the cohort of 433 subjects, called Model Population (MP). Each subject in the MP has complete four-digit HLA class I genotype (all six alleles) information available. The MP was assembled from three sources: (i) 270 subjects from the HapMap collection, including 90 Yoruban, 90 European, 45 Chinese, and 45 Japanese subjects [124], (ii) 67 subjects from the European Searchable Tumour Line Database (ESTDAB) database [125], including subjects from US, Canada, Australia, and New Zealand, and (iii) 96 subjects from the HIV database [126].

Epitope predictions were performed using Immune Epitope Database (IEDB) recommended setting that uses consensus approach [127,128]. The vaccine antigens were scanned with overlapping 9-mer peptides to identify epitopes that bind to any of a MP-subject’s six HLA class I alleles. These predictions were performed for each of the 433 subjects in the MP.

During the in silico modelling, predicted frequency of vaccine-specific HLA-binding epitopes were used to calculate the in silico IRRs for the MP (see also Table 2):

In Silico IRR (1 × HLA): the percentage of subjects in the MP with ≥ 1 vaccine-specific epitope binding to at least one autologous HLA class I allele.

In Silico IRR (2 × HLA): the percentage of subjects in the MP with ≥ 1 vaccine-specific epitope binding to at least two autologous HLA class I alleles.

In Silico IRR (3 × HLA): the percentage of subjects in the MP with ≥ 1 vaccine-specific epitope binding to at least three autologous HLA class I alleles.

In Silico IRR (4 × HLA): the percentage of subjects in the MP with ≥ 1 vaccine-specific epitope binding to at least four autologous HLA class I alleles.

In Silico multi-epitope IRR (1 × HLA): the percentage of subjects with ≥ 2 vaccine-specific epitopes binding to at least one autologous HLA class I allele.

In Silico multi-epitope IRR (2 × HLA): the percentage of subjects with ≥ 2 vaccine-specific epitopes binding to at least two autologous HLA class I alleles.

In Silico multi-epitope IRR (3 × HLA): the percentage of subjects with ≥ 2 vaccine-specific epitopes binding to at least three autologous HLA class I alleles.

In Silico multi-epitope IRR (4 × HLA): the percentage of subjects with ≥ 2 vaccine-specific epitopes binding to at least four autologous HLA class I alleles.

In Silico multi-Ag IRR (1 × HLA): the percentage of subjects with ≥ 2 vaccine-specific epitopes originated from different protein antigens targeted by the vaccine and binding to at least one autologous HLA class I allele.

In Silico multi-Ag IRR (2 × HLA): the percentage of subjects with ≥ 2 vaccine-specific epitopes originated from different protein antigens targeted by the vaccine and binding to at least two autologous HLA class I allele.

In Silico multi-Ag IRR (3 × HLA): the percentage of subjects with ≥ 2 vaccine-specific epitopes originated from different protein antigens targeted by the vaccine and binding to at least three autologous HLA class I allele.

In Silico multi-Ag IRR (4 × HLA): the percentage of subjects with ≥ 2 vaccine-specific epitopes originated from different protein antigens targeted by the vaccine and binding to at least four autologous HLA class I allele.

When the vaccine was intended for a specific subpopulation (HLA preselection), the MP was also stratified to the same specific subgroup (e.g., only HLA-A*0201 positive patients were enrolled, see Table 1). When the immunogenicity of a multi-peptide vaccine was measured and published per peptide, the in silico IRRs were also determined per peptide. When there were more than one study published for the same vaccine with the same HLA restriction, the cohorts of the studies were combined and RRs were calculated for the combined population (sum of responders for all trials/sum of total analyzed subjects in all trials, see Table A1).

These in silico IRRs were compared with the published IRRs and/or CRRs determined in the CTs.

### 2.3. Statistical Calculations

Representativeness of the MP was assessed by comparison of the summed allele set (152 different alleles) frequency of the MP with the summed frequency of the 4818 HLA alleles contained in the Catalog of common, intermediate and well-documented HLA alleles (CIWD) based on > 8 million subjects’ HLA background [129]. Epitope binding capabilities were compared with a 16,000 subject cohort (National Marrow Donor Program, NMDP cohort, see below) and were evaluated as follows: from the collected 94 CTs the 11 most frequently used target proteins were selected, which together spanned 5434 amino acids in length and included 5346 possible 9-mers. For both the MP and the NMDP cohort for each protein’s each amino acid the proportion of subjects who are able to bind an epitope (9-mer) starting at that position with ≥ 1, ≥ 2, ≥ 3, ≥ 4, ≥ 5 or all six HLA alleles were determined. For each HLA cut-off these frequencies for each amino acid position (MP versus NMDP cohort) were plotted. Correlation was determined by Pearson correlation coefficient (*r*), and statistical significance was computed following the Student’s t-distribution with degree of freedom *n*−2, with a significance threshold of *p* < 0.05. Epitope predictions were performed as described for the in silico trial [127,128].

The 16,000 subjects’ (NMDP cohort) HLA genotype data were obtained from the US National Marrow Donor Program [130]. This cohort of US origin covered 16 ethnic groups, with 1000 subjects in each: African, African American, Asian Pacific Islander, Filipino, Black Caribbean, Caucasian, Chinese, Hispanic, Japanese, Korean, Native American Indian, South Asian, Vietnamese, US, Mideast/North coast of Africa, Hawaiian, and other Pacific Islander.

For the measured and predicted response rates, correlations were assessed using the Pearson correlation coefficient (*r*), measuring linear correlation between two variables. A general trend line was used to compute confidence interval bands with level 0.95 probability and to predict interval bands with level of 0.95 probability. A perpendicular line was used to show the trend between the predicted and clinical outcome. This linear regression is based on the line that has the minimum perpendicular distance-squares from the points. Statistical significance was computed following the Student’s t-distribution with degree of freedom *n*−2.

Pairwise comparison of measured and predicted response rates were done using an online tool that is based on the “*n*−1” Chi-squared test as recommended by others [131,132,133,134]. Each data pair (measured and predicted response rate) were separately entered into the calculator together with the respective sample sizes. Difference between a measured and predicted data pair was considered significant when *p* < 0.05.

Receiver operating characteristic (ROC) area under the curve (AUC) was calculated based on the traditional 2 × 2 contingency table assembled using the following assumptions: (1) to obtain a binary classification, AUC was calculated for each IRR in the range of 30–80% to avoid imbalanced dataset, (2) in silico IRR data points were classified as true negative (TN), true positive (TP), false positive (FP), false negative (FN) based on the threshold, (3) sensitivity (TP/(TP + FN)) and specificity (TN/(TN + FP)) were calculated based on the 2 × 2 contingency table. To obtain the ROC curve, sensitivity was plotted against the 1-specificity and the AUC was calculated [135].

## 3. Results

### 3.1. Preselection of HLA-Matched Subjects Does Not Improve Response Rate Obtained in Clinical Trials

**Figure 1:**
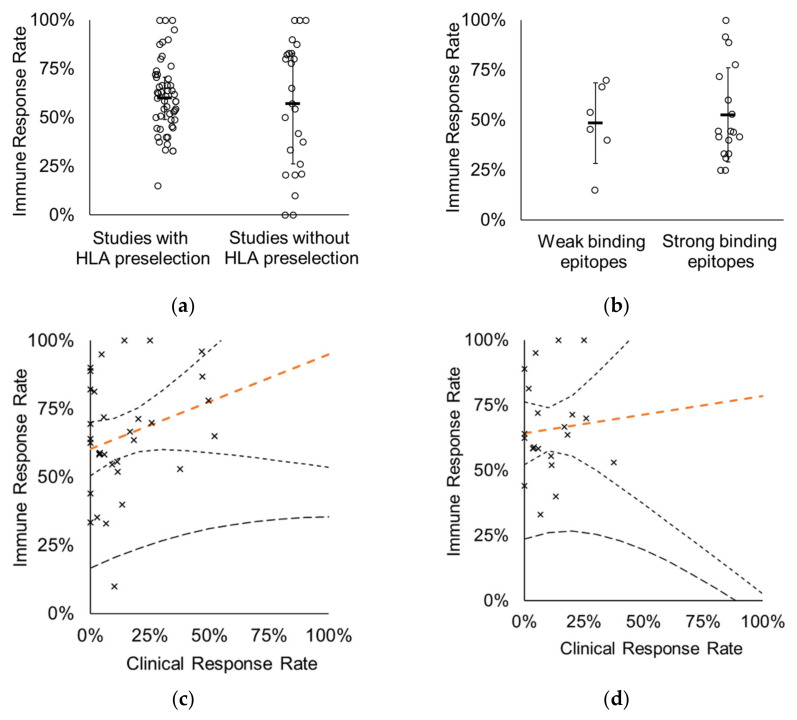
Immunogenicity of cancer vaccines is not predictive for efficacy. (**a**) IRR of CTs with (*n* = 52) or without (*n* = 25) HLA preselection of subjects. Medians (marked with horizontal line) were 60% and 57%, respectively (*p* = 0.4490). Each mark represents one CT. Error bars represent the standard deviation of the datasets. (**b**) IRR of CTs does not correlate with predicted binding affinity of vaccine epitopes. Strong (*n* = 18) and weak (*n* = 6) binding epitopes were grouped based on the predicted binding affinity to the HLA allele used for subject preselection. Strong binders are epitopes with < 2 IEDB percentile rank (*p* = 0.6657). Each mark represents one peptide. (**c**) IRR of vaccines does not correlate with clinical responses (*r* = 0.2594, *p* = 0.1495). Forty-two CTs of 33 vaccines were used in the analysis. (**d**) IRR of vaccines does not correlate with CRR in CTs where subjects are preselected based on HLA (*r* = 0.0782, *p* = 0.7293). Twenty-nine CTs of 28 vaccines were used in the analysis. Orange dashed line: perpendicular trend line with 95% confidence interval bands (95% prediction interval band is shown by thicker dashed line). Each mark represents one CT.

To study the parameters likely affecting the IRR and CRR of CTs, a meta-analysis of the immunological and clinical results reported in 94 CTs involving 2338 subjects treated with 64 immunotherapeutic vaccines targeting 88 different antigens were performed (Table 1 and Table A1). No significant difference was found between the IRRs of CTs preselecting the trial subjects based on their HLA alleles (*n* = 52 CTs) or accepting “all-comers” (*n* = 25 CTs) without HLA determination (median 60% vs. 57%, *p* = 0.4490) (Figure 1a).

This suggests that the presence of a matching HLA allele does not ensure the generation of CD8^+^ T-cell responses (immune responses) upon vaccination, thus it is not a valid predictor. In order to investigate whether the predicted binding affinity of an epitope included in a vaccine has major impact on the IRR, we selected those CTs where the CD8^+^ T-cell responses were reported for individual short peptides (9- or 10-mers) and the CT included the HLA preselection of the subjects. Fourteen CTs were eligible for such analysis, conducted with 13 vaccines covering a total of 24 peptides. No significant difference was found between the IRRs of strong binder (< 2 percentile rank) and weak binder (> 2 percentile rank) vaccine epitopes (average 53% and 49%, respectively, *p* = 0.6657) (Figure 1b). Due to the high standard deviation in both groups, applying more strict thresholds for the separation of strong- and weak-binder epitopes (at < 0.5 or < 1 percentile rank) also results in non-significant differences between the IRRs of the two groups (data not shown). This result supports the earlier finding that not only those epitopes are able to elicit immune response which are predicted as strong binders [30].

As expected, there was no correlation between the IRR and CRR reported for the studies (*r* = 0.2594), nor for the trials employing HLA preselection (*r* = 0.0782) (Figure 1c,d).

### 3.2. Characterization of the In Silico Model

#### 3.2.1. HLA Allele Frequency Analysis

Based on the results obtained in Figure 1a the criterion of a single HLA-match does not seem to be sufficient to predict the immunogenicity of vaccines. To overcome this we hypothesized that all six HLA alleles of a person could contribute to the generation of in vitro measured CD8^+^ T-cell responses, not only one of them. Therefore, the model was built on complete HLA genotype of individuals allowing to study the effect of the combination of all six HLA class I alleles. Since complete HLA genotype data of the subjects participating in the CTs were not available, a model cohort (MP) was built of 433 subjects with four-digit HLA class I genotype covering multiple ethnicities (see Materials and Methods). The representativeness of the MP was assessed by comparing the HLA allele coverage to the latest collection of allele frequencies included in the Catalog of common, intermediate and well-documented alleles (CIWD), which was compiled based on >8 million subjects’ HLA background [129]. The summed frequency of the 4818 HLA class I alleles included in the CIWD is considered as 1.00 (or 100%). Compared to this, the 152 HLA class I alleles covering the 433 subjects in the MP (Table A2) have a summed frequency of 0.974 (or 97.4%). This means that these 152 alleles are the most frequently occurring globally, and the remaining 4666 alleles are rare alleles (2.6%). Specific HLA-selected subpopulations of the MP used in the study also reach at least 89% coverage calculated following the same methodology (Table S1). Therefore, the likelihood that a person in a CT would have one or more of the rare alleles not covered by this set is low.

#### 3.2.2. Epitope-Binding Capabilities

**Figure 2:**
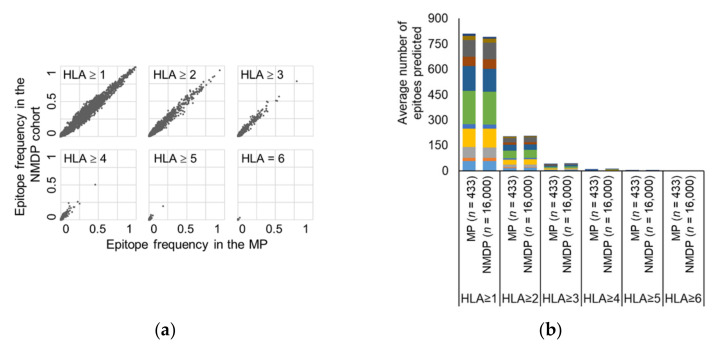
Representativeness of the in silico model cohort (MP) demonstrated by the correlation of epitope-binding capabilities with the NMDP cohort of 16,000 subjects. (**a**) Frequency of MP versus NMDP cohort subjects predicted to bind 9-mers of the 11 protein antigens for the six different cut-offs: HLA ≥ 1 alleles (*r* = 0.9897, *p* < 10^−102^), HLA ≥ 2 alleles (*r* = 0.9903, *p* < 10^−102^), HLA ≥ 3 alleles (*r* = 0.9849, *p* < 10^−102^), HLA ≥ 4 alleles (*r* = 0.9611, *p* < 10^−102^), HLA ≥ 5 alleles (*r* = 0.9401, *p* = 4.7 × 10^−102^), or HLA = 6 alleles (*r* = 0.8742, *p* = 1.8 × 10^−39^). Each point represents a possible 9-mer epitope of the selected 11 protein antigens. (**b**) Average number of epitopes predicted for the six different cut-offs: binding to ≥ 1, ≥ 2, ≥ 3, ≥ 4, ≥ 5, or =6 autologous HLA alleles for the 11 proteins; MAGE-A3 (■), survivin (■), WT1 (■), GP100 (■), NY-ESO-1 (■), HER2 (■), MUC1 (■), P53 (■), Tyrosinase (■), HPV-16 E6 (■) and HPV16-E7 (■).

Since the aim was to consider the combination of HLA alleles within a person, the representativeness of the MP was assessed also on this level. The search for databases or publications to find reference data for frequent HLA allele combinations/HLA genotypes was unsuccessful, thus as reference population a large cohort of 16,000 subjects with complete HLA genotype (NMDP cohort, see Methods) was chosen. As a comparison to the MP, this cohort has 497 different HLA class I alleles with a summed frequency of 0.998 (similar analysis as above). To prove that the epitope-binding capabilities of the HLA allele combinations of MP correlate with the ones of the large 16,000 subject population, epitope mapping was performed for the 11 most frequently used vaccine proteins based on the collected dataset. For all 11 proteins’ each possible 9-mer the frequency of subjects was determined in the MP and in the NMDP cohorts, who were predicted to bind the specific epitope by at least 1, 2, 3, 4, 5, or all 6 of their HLA alleles. Figure 2a shows the correlation plots obtained for the six HLA cut-offs, each demonstrating strong correlation (*p* < 10^−39^ and r of 0.874–0.991) between the epitope-binding capability of the MP and the NMDP cohorts.

The average number of epitopes binding to at least 1, 2, 3, 4, 5, or all 6 HLA alleles for these 11 proteins was also compared between the two populations, which was found to be similar (Figure 2b). This analysis also shows that a fraction of epitopes (25%–26%) are able to bind multiple (≥ 2) HLA alleles of a subject, potentially supporting the relevance of our hypothesis. The average number of epitopes for a person that bind at least 5 HLA alleles is so small (< 0.3 epitopes) that the ≥ 5 HLA and = 6 HLA cut-offs were not further investigated in the present study. Based on these results the MP was considered representative in terms of HLA allele frequency and epitope-binding capability of the HLA-sets (HLA genotypes) for the cohorts involved in the CTs of this study.

### 3.3. In Silico IRRs in the MP Correlate with IRRs Measured in CTs

#### 3.3.1. Correlation Analysis between In Silico and Measured IRRs

**Figure 3:**
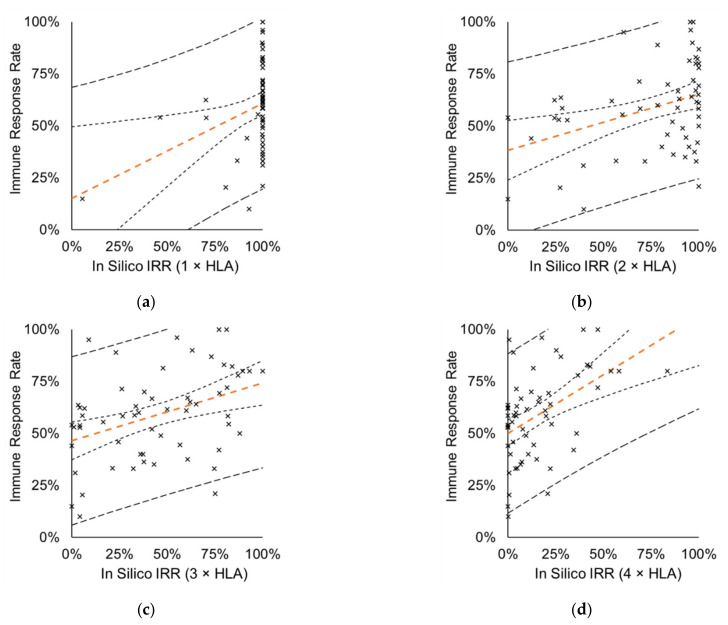
Correlation analysis between in silico and measured IRRs. In silico prediction was based on the proportion of subjects in the MP having at least one vaccine-specific epitope that is (**a**) restricted to ≥ 1 autologous HLA class I allele (*r* = 0.3225, *p* = 0.0127), (**b**) restricted to ≥ 2 autologous HLA class I alleles (*r* = 0.3763, *p* = 0.0033), (**c**) restricted to ≥ 3 autologous HLA class I alleles (*r* = 0.4015, *p* = 0.0016), (**d**) restricted to ≥ 4 autologous HLA class I alleles (*r* = 0.4780, *p* = 0.0001). Analysis included 59 data pairs covering 79 CTs with 55 vaccines. Orange dashed line: perpendicular trend line with 95% confidence interval band (95% prediction interval band is shown by thicker dashed line).

Next, we aimed to demonstrate that predicted multiple autologous HLA allele-binding epitopes better characterize the IRR of therapeutic vaccines. To achieve this, in silico IRRs were determined by calculating the proportion of subjects in the MP having at least one vaccine-specific epitope that is predicted to bind ≥ 1, ≥ 2, ≥ 3 or ≥ 4 autologous HLA class I alleles (In Silico IRR (1 × HLA), In Silico IRR (2 × HLA), In Silico IRR (3 × HLA), In Silico IRR (4 × HLA), respectively, see also Materials and Methods and Table 2). Of note, IRR of the clinical studies is usually reported for T-cell responses measured against at least one epitope (a peptide or a peptide pool), therefore this criterion was used in this study as well. In the analysis 79 CTs conducted with 55 vaccines, resulting in 59 data points were included. Analysis revealed that single HLA allele binding epitopes (cut-off HLA ≥ 1) highly overestimated the measured IRRs as more than 80% of the CTs were predicted to have at least one epitope restricted to at least one HLA allele of each of the 433 subjects (100% In Silico IRR (1 × HLA)), therefore the correlation was weak (*r* = 0.3225, *p* = 0.0127) (Figure 3a).

A similar shift of points can be observed for In Silico IRR (2 × HLA) (*r* = 0.3763, *p* = 0.0033) with a less marked pattern (Figure 3b). In Silico IRR (3 × HLA) shows a more balanced distribution of the data pairs indicating substantial relationship between the frequency of the epitopes restricted to at least three autologous HLA alleles and IRRs measured in the CTs (*r* = 0.4015, *p* = 0.0016) (Figure 3c). Similarly, for the In Silico IRR (4 × HLA) moderate correlation (*r* = 0.4780, *p* = 0.0001) was observed with the IRRs (Figure 3d) and a tendency to underestimate IRRs (points shifted towards left), opposite to In Silico IRR (1 × HLA) and In Silico IRR (2 × HLA).

**Figure 4:**
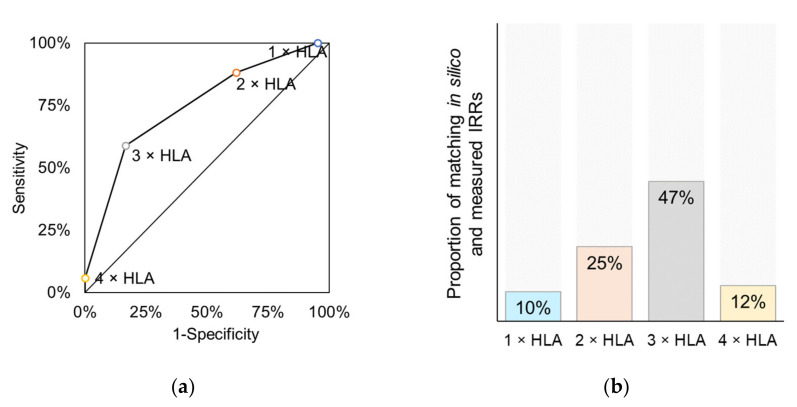
Performance evaluation of the in silico trials. (**a**) Example of a Receiver Operating Characteristic (ROC) curve of the in silico IRR predictions when the success threshold is considered as ≥ 70% IRR (AUC = 0.75) (related to Table S2). (**b**) Pairwise Chi square analysis of measured and predicted IRRs; bars represent the proportion of the analyzed data pairs where difference was not significant (*p* > 0.05). Analyzed dataset was the same as used for Figure 3 (59 data pairs covering 79 CTs with 55 vaccines).

ROC curve analysis confirmed the association between in vitro-measured IRR and the frequency of the multiple autologous allele-binding epitopes (in silico IRRs). The area under the ROC curve (AUC) for each IRR threshold (in the 30% to 80% interval) was in the range of 0.63–0.79, indicating fair/good accuracy [136] of the prediction independent of chosen IRR thresholds (Figure 4a and Table S2).

#### 3.3.2. Pairwise Comparison of In Silico and Measured IRRs

Pairwise Chi square analysis revealed that In Silico IRR (1 × HLA) correctly predicted IRR for only 10% (6/59) of the analyzed data pairs (no significant difference between measured and in silico predicted IRR values, *p* > 0.05), while this proportion was the highest, 47% (28/59), for In Silico IRR (3 × HLA) (Figure 4b).

Pairwise analysis was also performed by grouping the data pairs based on vaccine type: peptide vaccines (46 data pairs), dendritic cell (DC) vaccines (4 data pairs), and nucleic acid vaccines, covering plasmid DNA, viral vector and mRNA vaccines (9 data pairs). As expected based on the majority of peptide vaccines in the dataset, the proportions of matching results obtained for peptide vaccines were similar with the combined dataset shown in Figure 4b: 13%, 33%, 43% and 7% for the HLA thresholds ≥ 1–4, respectively (Figure S1). The separate evaluation of DC vaccines and nucleic acid-based vaccines also show the superiority of HLA ≥ 3 threshold; however these results should be interpreted with caution due to the low number of data pairs (Figure S1).

These results suggest that the multiple autologous HLA allele-binding concept outperforms the conventional single-HLA allele-binding approach and the in silico IRRs as determined by the model are able to retrospectively estimate immunogenicity of the therapeutic vaccines.

### 3.4. Relationship between Immune- and Clinical Response

#### 3.4.1. Vaccines Targeting Multiple Epitopes

**Figure 5:**
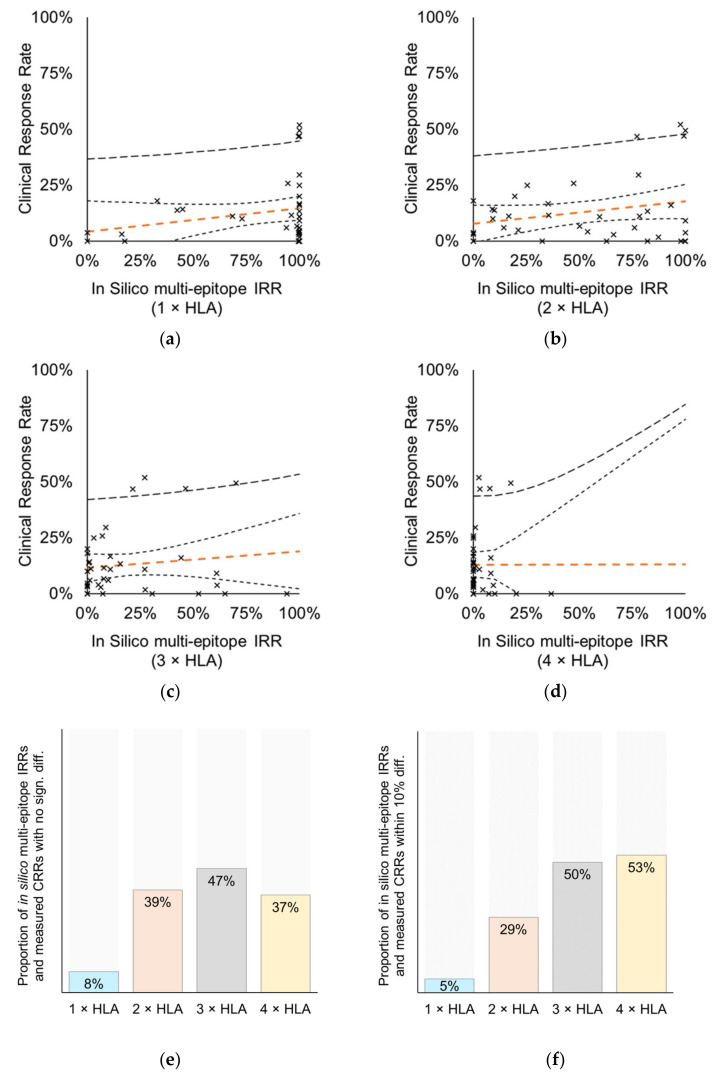
Correlation analysis between CRRs measured by CTs and in silico multi-epitope IRRs with cut-offs HLA ≥ 1, ≥ 2, ≥ 3, or ≥ 4 in the MP. Analysis included 49 CTs with 31 vaccines, total of 38 data points. Prediction of multi-epitope IRRs required the presence of at least two vaccine-specific epitopes that are (**a**) restricted to ≥ 1 autologous HLA class I allele (*r* = 0.2233, *p* = 0.1777), (**b**) restricted to ≥ 2 autologous HLA class I alleles (*r* = 0.2520, *p* = 0.1269), (**c**) restricted to ≥ 3 autologous HLA class I alleles (*r* = 0.1253, *p* = 0.4536), (**d**) restricted to ≥ 4 autologous HLA class I alleles (*r* = 0.0003, *p* = 0.9985) of the subjects in the MP, respectively. Orange dashed line: perpendicular trend line with 95% confidence interval band (95% prediction interval band is shown by thicker dashed line). (**e**,**f**) Pairwise Chi square analysis of measured CRRs and predicted multi-epitope IRRs; bars represent the proportion of the analyzed data pairs where difference was (**e**) not significant (*p* > 0.05), or (**f**) within 10%.

As previously suggested, subjects having broader immune responses (against multiple vaccine-specific epitopes) may experience clinical benefit, therefore the relationship between the in silico IRRs and clinical responses was next examined. As a measure of vaccines’ ability to induce broad immune responses, the in silico IRRs against multiple epitopes were calculated for each HLA threshold; the percentage of subjects in the MP with ≥ 2 vaccine-specific epitopes binding to ≥ 1, ≥ 2, ≥ 3 or ≥ 4 autologous HLA class I alleles (In silico multi-epitope IRR (1 × HLA), In silico multi-epitope IRR (2 × HLA), In silico multi-epitope IRR (3 × HLA), In silico multi-epitope IRR (4 × HLA), respectively). This analysis included 38 data pairs of 49 CTs conducted with 31 vaccines (Table A1). Using the Pearson correlation analysis no correlation was found between CRR and any of the in silico multi-epitope IRRs (Figure 5a–d).

Again, the datasets were analyzed pairwise for each HLA threshold to make a point by point comparison of predicted and measured data. When assessing the significance of the differences obtained for the data pairs, the most non-significantly different pairs (18/38, 47%) were obtained again with the ≥ 3 HLA threshold (Figure 5e). The other multi-HLA cut-offs also performed fairly in predicting CRRs (39% for 2 × HLA and 37% for 4 × HLA). Specifically, ≥ 50% of the data pairs were found to be within 10% difference for HLA ≥ 3 and HLA ≥ 4 cut-offs (Figure 5f). While for the single HLA allele-restricted epitopes (1 × HLA) the in silico multi-epitope IRRs matched the CRRs in only 8% of cases (Figure 5e).

Similar to the IRR analysis, we investigated the pairwise agreement in subgroups of vaccine types: peptide vaccines (30 data pairs), DC vaccines (3 data pairs), and nucleic acid vaccines, covering plasmid DNA and viral vector vaccines (6 data pairs). Again because of the dominance of peptide vaccines among the data pairs the result was comparable to the combined analysis shown in Figure 5e, and reinforced for other vaccine types, too (Figure S2).

#### 3.4.2. Vaccines Targeting Multiple Protein Antigens

**Figure 6:**
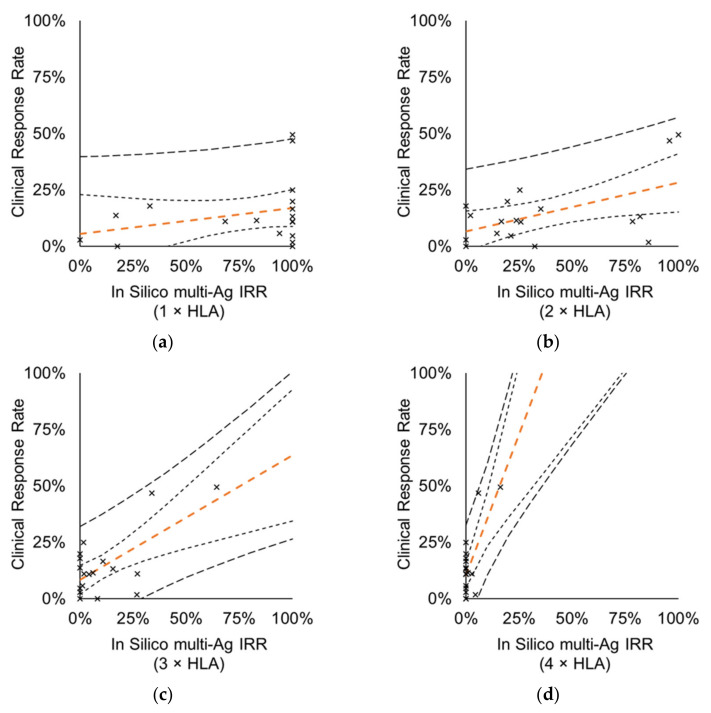

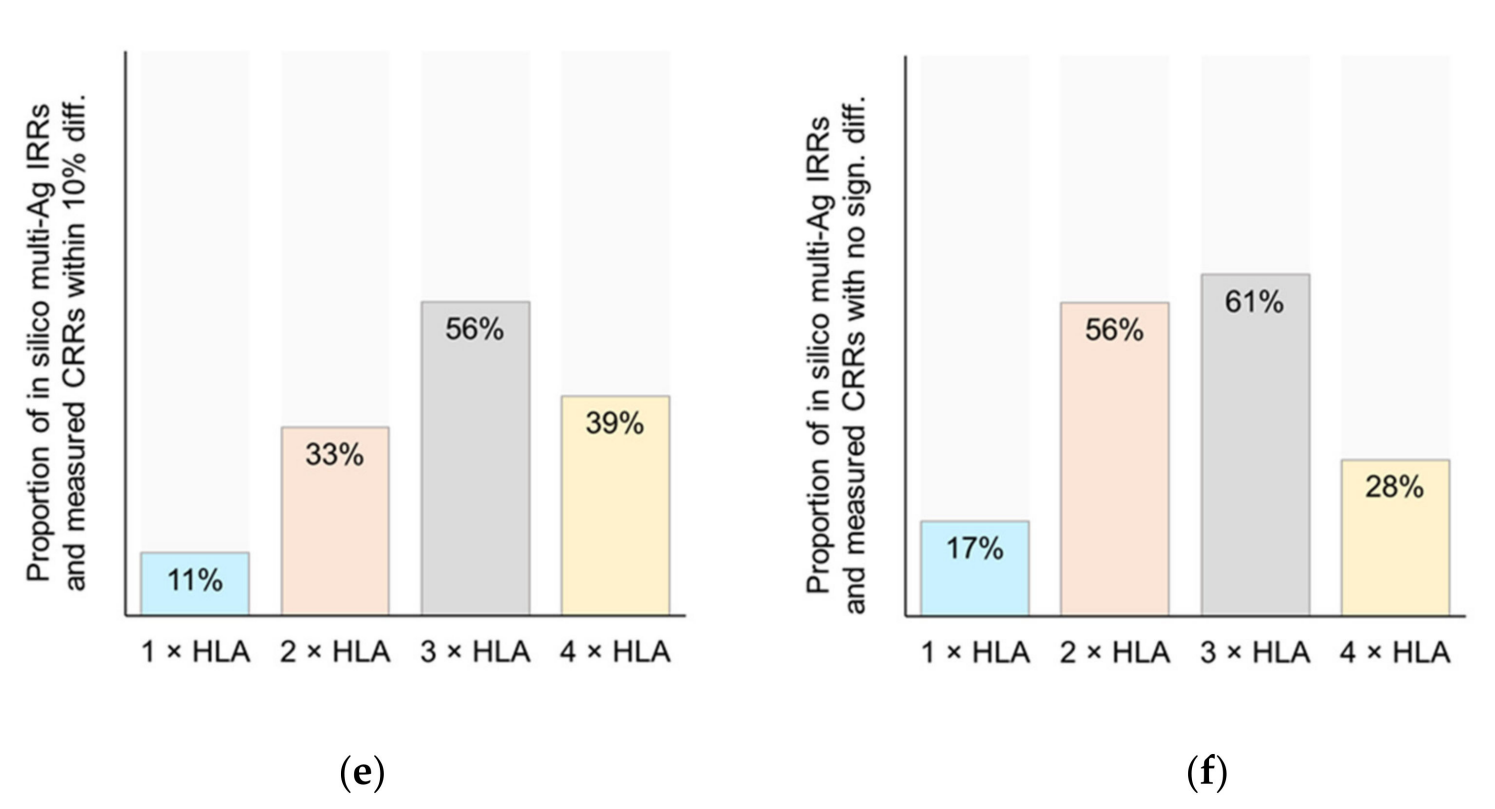
Correlation between CRRs measured by CTs and in silico multi-Ag IRRs. Analysis included 23 CTs with 16 vaccines, total of 18 data points. Prediction of multi-Ag IRRs is based on the frequency of subjects in the MP with at least two vaccine-specific epitopes originated from different protein antigens that are (**a**) restricted to ≥ 1 autologous HLA class I allele (*r* = 0.2865, *p* = 0.2491), (**b**) restricted to ≥ 2 autologous HLA class I alleles (*r* = 0.5355, *p* = 0.0220), (**c**) restricted to ≥ 3 autologous HLA class I alleles (*r* = 0.6709, *p* = 0.0023), (**d**) restricted to ≥ 4 autologous HLA class I alleles (*r* = 0.7116, *p* = 0.0009). Orange dashed line: perpendicular trend line with 95% confidence interval band (95% prediction interval band is shown by thicker dashed line). (**e**,**f**) Pairwise analysis of measured CRRs and predicted multi-Ag IRRs. Bars represent the proportion of the analyzed data pairs where difference was (**e**) within 10%, (**f**) not significant (*p* > 0.05).

Another measure for the breadth of immune responses may be the percentage of subjects with ≥ 2 vaccine-specific epitopes originated from different protein antigens targeted by the vaccine, i.e., multi-antigenic IRR. These in silico IRRs were computed for each HLA threshold (In silico multi-Ag IRR (1 × HLA), In silico multi-Ag IRR (2 × HLA), In silico multi-Ag IRR (3 × HLA), In silico multi-Ag IRR (4 × HLA)) and compared with the CRRs measured in the CTs (Figure 6a–d).

Eighteen data pairs of 23 CTs conducted with 16 vaccines were eligible for such analysis (Table A1). There was a good/strong correlation for each of the models where multiple HLA class I allele binding was required (*r* = 0.5355, *r* = 0.6709 and *r* = 0.7116 for 2 × HLA, 3 × HLA, and 4 × HLA, respectively), but not for single HLA allele binding epitopes (*r* = 0.2865, *p* = 0.2491) (Figure 6b–d). A pairwise comparison of measured and predicted data showed (Figure 6e) that 56% of the data pairs are within 10% difference and 61% differs non-significantly (*p* > 0.05, Figure 6f) for epitopes restricted to ≥ 3 autologous HLA class I alleles. All other HLA thresholds perform worse, especially the single HLA (1 × HLA) threshold where only 17% of data pairs matched. For the vaccines targeting multiple antigens no subgroup pairwise analysis of different vaccine types was performed, because 16/18 data points were peptide vaccines, with one plasmid DNA-based and one DC vaccine.

**Figure 7:**
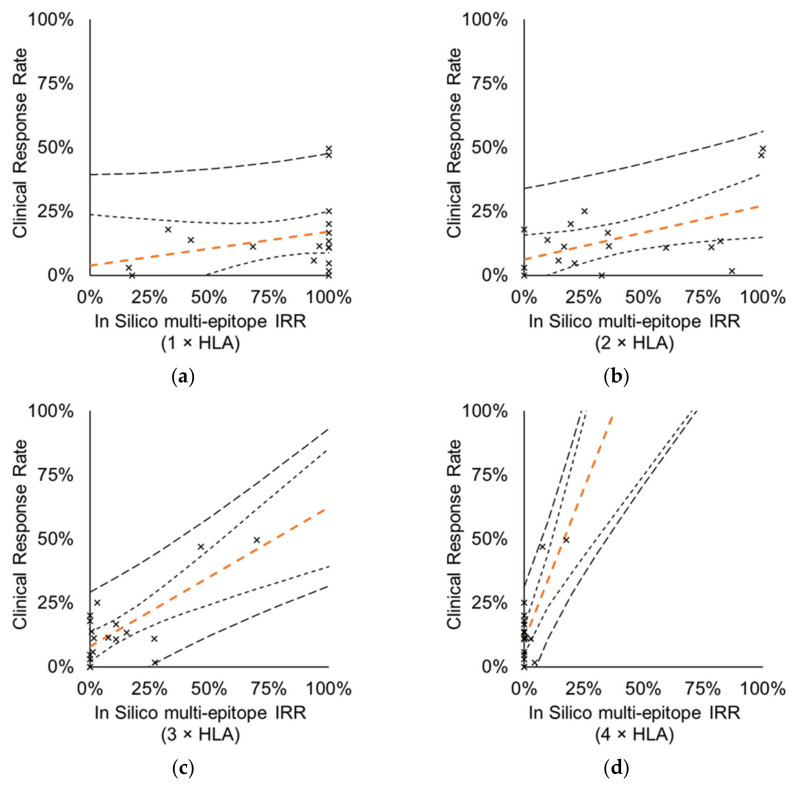
Correlation between CRRs measured by CTs and in silico multi-epitope IRRs of multiantigen-targeting vaccines. Analysis included 23 CTs with 16 vaccines, total of 18 data points. Prediction of multi-epitope IRRs is based on the frequency of subjects in the MP with at least two vaccine-specific epitope that are (**a**) restricted to ≥ 1 autologous HLA class I allele (*r* = 0.2909, *p* = 0.2415), (**b**) restricted to ≥ 2 autologous HLA class I alleles (*r* = 0.5253, *p* = 0.0252), (**c**) restricted to ≥ 3 autologous HLA class I alleles (*r* = 0.7463, *p* = 0.0004), (**d**) restricted to ≥ 4 autologous HLA class I alleles (*r* = 0.7462, *p* = 0.0004). Orange dashed line: perpendicular trend line with 95% confidence interval band (95% prediction interval band is shown by thicker dashed line).

Of note, in the subgroup analysis of the 16 vaccines that target multiple antigens (analyzed above) in addition to the in silico multi-Ag IRRs, multi-epitope IRRs (Figure 7) also significantly correlated to CRR if ≥ 2 HLA alleles were considered (*r*(2 × HLA) = 0.5253, *r*(3 × HLA) = 0.7463, *r*(4 × HLA) = 0.7462).

These results suggest that polyclonality of the vaccine-induced CD8^+^ T-cell responses is important to achieve tumor cell killing and thus tumor shrinkage.

## 4. Discussion

Despite the many controversial but unexplained data obtained for the immunogenicity of cancer vaccines, it is currently thought that HLA plays a major role in the development of the immune responses. Therefore, we hypothesized that all HLA alleles (HLA genotype) of a subject regulate immune responses not only some specific alleles. To investigate this concept an in silico cohort of real subjects was assembled and characterized as covering 97.4% of the global HLA alleles and major ethnicities. This cohort was used to retrospectively model the immunogenicity of therapeutic vaccines by predicting the proportion of subjects who are able to present vaccine-specific epitopes bound to their ≥ 1, ≥ 2, ≥ 3 or ≥ 4 autologous HLA alleles. This study shows that conventional prediction of T-cell responses based on a single HLA-restricted epitope highly overestimates in vitro measured IRR of vaccine CTs, and thus fails as trial enrichment strategy as well [16]. The study suggests that HLA allele binding is a required but potentially not sufficient criteria for in vitro measured T-cell responses.

It is also suggested here that the subjects’ complete HLA genotype is a major determinant of vaccine responses. A positive relationship between the number of HLA alleles contributing to epitope binding and the IRR obtained in the studies is shown. We identified that ≥ 3 autologous HLA allele binding epitopes link the subjects’ HLA alleles with measured CD8^+^ T-cell responses and correctly predict the immunogenicity outcome for the majority of studies. In this study the relationship between multiple autologous HLA allele-binding epitopes and T-cell responses was obtained on population (CT) level. In some earlier studies of our group where the patients’ HLA genotype were available correlation was shown between measured T-cell responses and predicted epitopes that bind to ≥ 3 autologous-HLA alleles on individual level (we call these epitopes personal epitopes, or PEPIs). In HLA-genotyped COVID-19 convalescent subjects our group reported significant correlation of measured T-cell responses and predicted SARS-CoV-2-specific PEPIs, while no association with single HLA-restricted epitopes was found [137]. Similar observations were made for HLA-genotyped patients with (pre)malignant cancers who were treated with a Synthetic Long Peptide Vaccine encoding HPV16, where we have found 90% agreement between the measured CD8^+^ T-cell responses and the predicted PEPIs, but no correlation between single HLA-binding epitopes and T-cell responses [138]. Moreover, the magnitude of CD8^+^ T-cell responses measured by ELISPOT assay was significantly higher for PEPIs compared to non-PEPIs [137]. These results indicate that conventional prediction of single HLA-restricted epitopes highly overestimates T-cell responses (high false positive rates) and could explain the high clinical failure rates of vaccines that are matched to only a single HLA allele of patients [62,74,139], as well as the low specificity of predicted high affinity HLA class I binding neoepitopes [25,140,141].

The lack of correlation between individuals’ immune responses and objective clinical responses is the major source of the skepticism associated with cancer vaccines [1,2]. These observations are further supported by the present analysis showing no correlation between IRR and CRR using the dataset of 42 CTs of 33 vaccines. Therefore, not surprisingly, there was also no correlation between the predicted IRR (with at least one vaccine-specific epitope) and reported CRR for these studies. However, when predicting multi-epitope responses or multi-antigenic responses (epitopes derived from at least two different antigens) for the vaccines targeting multiple tumor antigens, significant correlation was found for all HLA ≥ 2 cut-off values but not with single HLA allele restricted epitopes. This result suggests that for IRRs reported with more stringent criteria (e.g., CD8^+^ T-cell responses against at least two epitopes instead of one) correlations with CRR could be likely observed. Association between clinical benefit and immune response against multiple tumor targets were reported for few CTs. For the IMA901 renal cell carcinoma vaccine the disease control rate was associated with vaccine-induced immune responses in the subpopulation of multi-peptide responders (T-cell responses to ≥ 2 vaccine peptides) [142]. Multi-peptide response was also associated with longer overall survival [5]. In another multi-peptide vaccine against glioma the investigators reported a similar observation: patients with positive ELISPOT responses to two or more antigens were more likely to have objective radiological responses than those who responded to only a single peptide [47].

The fact that both IRR and CRR could be predicted by multiple HLA allele-restricted epitopes suggests T-cell involvement. This phenomenon is supported by the observation that the expression of individual classical HLA class I loci (HLA-A, -B and -C) has been found balanced within each human tissue with the highest level in immune cells [143]. Consequently, the same epitope can be naturally presented by more than one autologous class I HLA allele, suggesting that the A, B, and C alleles each contribute to the activation of T-cells, and consequently the more T-cells are elicited by the vaccine, the more IFN-γ positive cells will be detected by the in vitro assays (i.e., ELISPOT). Moreover, multiple HLA allele restricted epitopes within a person may activate a broader repertoire of epitope specific T-cells with different T-cell receptor (TCR) clonotypes, thereby increasing the immunogenicity [12,13,14,25,144]. Furthermore, at the tumor side, the PEPIs might trigger more cytotoxic T-cell clones than epitopes restricted to a single HLA allele, as they could overcome the common tumor immune escape mechanism by HLA downregulation (thus less efficient epitope presentation) [145,146]. These results suggest that triggering multiple cytotoxic T-cell clones against (multiple) epitopes jointly presented by multiple HLA alleles on the surface of the tumor might be essential to achieve tumor shrinkage.

Our results confirm that there is a relationship between vaccine-induced immune responses and subsequent clinical responses, but only in a subgroup of subjects with a specific HLA genotype capable of presenting epitopes by their multiple HLA alleles. This is in good agreement with the recent finding that patients’ HLA class I genotype (HLA heterozygosity) influences response to checkpoint inhibitor therapy presumably due to efficient HLA presentation of tumor antigens triggering efficient CD8^+^ T-cell responses [147].

Objective tumor responses may depend on multiple variables (e.g., true expression of target antigens on the heterogeneous tumor) and definitely one of them is the generation of multi-targeted T-cell responses. Therefore, the design of therapeutic cancer vaccines should focus first of all on ensuring robust immune responses against the encoded (multiple) tumor targets in each subject. Preclinical animal models are indisputably important for the mapping and understanding of the mechanism of action of immunotherapeutics, however it is well-known that preclinical immunogenicity and efficacy does not correlate well with human results [148,149]. Therefore, a new in silico tool that could accurately predict the immunogenicity of therapeutic vaccines could bring a revolution to the development of cancer vaccines. Such a model should rely on subjects with complete HLA genotype rather than on single alleles. This could be used also for in silico epitope mapping in the design of the vaccines, to select the epitopes that are predicted to be immunogenic in the majority of subjects or ethnic populations.

This study has several limitations. The basic limitation is that the cohort used in this study is not the same as the CT populations. Although the HLA allele coverage compared to the CIWD database was shown to be similar, HLA-genotype of the individuals cannot be confirmed. Since HLA alleles have a major role in tumor surveillance, many groups explored and found associations of certain HLA alleles or haplotypes with cancers, including melanoma [150], breast cancer [151], colorectal cancer [152], head & neck cancer [153], cervical cancer [154], and ovarian cancer [155]. These studies revealed the increase or decrease in the frequency of the specific alleles, notably most of these associations were identified with HLA class II alleles. In a recent publication, Marty and co-workers reported their finding, that HLA class I genotype of cancer subjects shape their tumors’ mutational profile by eliminating through immunological reactions those neoepitopes that are highly presented by the HLAs [156]. These results however, support our finding. The lack of a reference dataset for frequent HLA allele combinations or HLA genotypes limits the unambiguous demonstration of representativeness for our MP. The model may be fine-tuned by the use of in silico populations assembled from HLA-genotyped subjects having the target disease, which could result in a more accurate estimation of clinical outcomes. Another limitation is that since the model is based on the genetic capability of a person to present epitopes and mount immune responses, it was not possible to address or incorporate the contribution of vaccine antigen type, formulation [157,158,159,160], route of administration [91], or dosing and schedule. A marginal limitation of the study is the variability of the immunoassays used for the determination of immune responders, which not only applies to the use of different assays but also varying thresholds or criteria for positivity. There is no gold standard or approved in vitro diagnostic device to measure vaccine induced T-cell responses, and standardization/validation of bioassays is often problematic even within the same laboratory [161,162,163]. This issue may contribute to the poor reproducibility of IRR across CTs. This was apparent in case of the p53 SLP70-248 vaccine CTs, where in one study none of the ten enrolled subjects had immune response but in other studies with comparable sample sizes the IRR was 88% and 100% (Table A1) [82,83,84]. If the true IRR was larger than 88%, then the probability of not detecting any immune response in ten subjects is extremely low (< 1.38 × 10^−9^). Of course, the small sample size of the CTs may also contribute to such issues. The other major limitation of the study is that the effect of previous treatments is not taken into consideration, however it may have huge impact, especially on the clinical response, when modulating tumor microenvironment [1,164,165,166,167]. When evaluating the correlations with clinical responses, we have to note the lack of CRR results published above 50%, which is a serious limitation of this analysis, and we think that the inclusion of successful trials (CRR > 50%) in our analysis would greatly improve the correlations.

## 5. Conclusions

This study shows that our in silico model together with the promiscuous autologous HLA allele binding epitope concept is able to estimate both the IRR and CRR of CTs. Moreover, to the best of our knowledge, for the first time in the literature correlation between IRR and CRR was found across multiple cancer vaccine studies. In all analyzed aspects predicted multiple HLA allele-binding epitopes outperformed the conventional single HLA-binding epitopes, which is potentially thought-provoking. The ability to predict the clinical outcome of therapeutic vaccine trials could expedite vaccine development by enabling the most immunologically powerful vaccine candidates to be selected for clinical testing. This could increase the likelihood of clinical success and reduce the need for large studies. Consequently, the clinical development time and cost of therapeutic vaccines could be reduced substantially.

## 6. Patents

The Authors filed patent application (WO2018/158456) resulting from the work reported.

## Figures and Tables

**Table 1:** Publications/Studies Included in the meta-analysis.

Immunotherapy	Indication	Type	HLA Restriction	Ref.
9-peptide breast cancer vaccine	BC	Peptide	HLA-A1, -A2, or -A3	[37]
AFP-derived peptides	aHCC	Peptide	A24	[38]
Antigen-pulsed DC vaccine	PAdC	pDC	A02	[39]
CD34 DC vaccine	MEL	pDC	A02:01	[40]
CV9103 mRNA vaccine	PC	mRNA	no	[41,42]
DCCP peptide vaccine	MEL	pDC	A24 or A02	[23]
DPX0907 peptide vaccine	BC, OC, PC	Peptide	A02	[43,44]
Elenagen pDNA vaccine	BC, CRC, KC, LC, OC, MEL	pDNA	no	[45]
EMD640744 peptide vaccine	S.tumors	Peptide	HLA-A1, -A2, -A3, -A24, -B7	[22]
Five-peptide cancer vaccine	S.tumors	Peptide	A24:02	[46]
GAA peptides vaccine	glioma	Peptide	A02	[47,48]
GL-0817 (MAGE-A3 Trojan)	SCCHN	Peptide	no	[49]
	MM			[50]
	mSCCHN			[51]
Glypican-3 peptide vaccine	pediatric tumors	Peptide	A02 or A24:02	[52]
	HCC	Peptide	A02 or A24:02	[53]
GVX301 peptide vaccine	PC, KC	Peptide	A2	[54]
HER1 vaccine	PC	Peptide	no	[55]
Her2 B-cell peptide vaccine	S.tumors	Peptide	no	[56]
Her2/neu peptide vaccine	BC, OC	Peptide	A02	[57]
HIVIS	HIV positivity	pDNA	no	[58]
HPV-SLP	VIN3	Peptide	no	[59]
	CC			[60]
	OrC, CC, AC			[61]
ICT107	GB or BSG	Peptide	A1 or A2	[62]
IDO and survivin peptide vaccine	mMEL	Peptide	A02	[63]
IDO long peptide vaccine	adv. MEL	Peptide	no	[64]
IMA901	RCC	Peptide	A02	[5]
	mRCC			
IMA950	AC, glioma	Peptide	A02	[65]
	AC, ODG			[66]
	GB			[67]
ImMucin	MM	Peptide	no	[68]
	CRC, LC, PC, TT			[69]
IMP321/LAG-3Ig + peptides vaccine	mMEL	Peptide	A02	[70]
INGN-225 p53 vaccine	SCLC	VV	A02	[71]
KIF20A-66 peptide vaccine	met. PaC	Peptide	A24:02	[72]
KRM-20 vaccine	PC	Peptide	A24	[73]
MART-1 Peptide Vaccine	MEL	Peptide	A02	[74]
Melanoma peptide vaccine	MEL	Peptide or pDC	A1, -A2 or -A3	[75]
MELITAC 12.1	MEL	Peptide	A1, -A2, or -A3; HLA-DR1, -DR4, -DR11, -DR13, or -DR15	[24]
Multiepitope peptide vaccine	CC	Peptide	A24:02	[76]
NY-ESO-1 OLP	MEL, EC, LC	Peptide	no	[77]
	OC, FTC, PerC			[78]
NY-ESO-1f	EC, GC, NSCLC	Peptide	no	[79]
OCV-C02 vaccine	CRC	Peptide	A24:02	[80]
p53 SLP70-235	CRC	Peptide	no	[81]
p53 SLP70-248	CRC	Peptide	no	[82]
	EOC			[83,84]
p53MVA vaccine	EOC, FTC, PerC	VV	no	[85]
PepCan	CIN2/3	Peptide	no	[86]
Peptide cocktail therapy	PaC	Peptide	A24:02 w/o	[87]
Peptide vaccine 1	CRC	Peptide	A24:02	[88]
Peptide vaccine 2	HNSCC	Peptide	A24:02	[89]
Peptide vaccine 3	AML	Peptide	A02:01	[90]
pNGVL4a-CRT/E7(detox) vaccine	CIN2/3	pDNA	No	[91]
PR1 Peptide Vaccine	AML, MDS, CML	Peptide	A02	[21]
ProstVac	PC	VV	A02	[92,93,94,95,96,97]
PSMA-Survivin pulsed autologous DC vaccine	PC	pDC	A02:01	[98]
PVX-410 peptide vaccine	MM	Peptide	A02	[99]
RHAMM-R3	AML	Peptide	A02	[100]
S-288310 peptide vaccine	BLC	Peptide	A24:02	[101]
StimuVax	NSCLC	Peptide	no	[102]
SVN-2B peptide vaccine	GIC, BDC, PaC	Peptide	A24:02	[103]
Synchrotope TA2M	MEL	pDNA	A02, but pts were not typed	[104]
TARP vaccine	PC	Peptide or pDC	A02:01	[105]
TG4010	cancer	VV	no	[106]
	PC		no	[107]
	RCC		no	[108]
	NSCLC		no	[109]
TSPP peptide vaccine	cancer	Peptide	no	[110]
	mCRC			[111]
VGX-3100	CIN2/3	pDNA	no	[112,113]
Vx-001	S.tumors	Peptide	A02:01	[114,115]
	NSCLC			[116,117]
WT1 vaccine (1)	glioma	Peptide	A24:02	[118]
	PaC	pDC	A24:02	[119]
	BC, OC, GC			[120]
WT1 peptide vaccine	AML MDS	Peptide	A02	[121]
WT1 peptides	PaC	Peptide	A02:01 or A24:02	[122]
WT1 vaccine (2)	mPM	Peptide	no	[123]

Abbreviations: DC: dendritic cell, AFP: Alpha-fetoprotein, HPV: human papilloma virus, SLP: synthetic long peptide, IDO: Indoleamine 2,3-Dioxygenase, MART1: melanoma antigen recognized by T-cells 1, NY-ESO-1: New York Esophageal Squamous Cell Carcinoma-1, MVA: Modified vaccinia Ankara, PSMA: prostate-specific membrane antigen, RHAMM: receptor for hyaluronic acid mediated motility, TARP: T-cell receptor alternate reading frame protein, TSPP: Thymidylate synthase poly-epitope peptide, WT1: Wilms Tumor Protein 1, PAdC: adenocarcinoma of the pancreas, aHCC: advanced hepatocellular carcinoma, PC: prostate cancer, BC: breast cancer, OC: ovarian cancer, EOC: epithelial ovarian cancer, CRC: colorectal cancer, KC: kidney cancer, pDNA: plasmid DNA, mSCCHN: metastatic squamous cell carcinoma of the head and neck, MM: multiple myeloma, RCC: renal cell carcinoma, CC: cervical cancer, VIN: vaginal intraepithelial neoplasia, GB: glioblastoma, AC: astrocytoma, ODG: oligodendroglioma, SCLC: small cell lung cancer, NSCLC: non-small cell lung cancer, PaC: pancreatic cancer, MEL: melanoma, BSG: brainstem glioma, TT: testicular tumors, EC: esophageal cancer, FTC: fallopian tube carcinoma, PerC: carcinoma of the peritoneum, GC: gastric cancer, CIN: cervical intraepithelial neoplasia, AML: acute myeloid leukemia, MDS: myelodysplastic syndrome, BLC: bladder cancer, GIC: gastrointestinal cancer, BDC: bile duct cancer, OrC: oropharyngeal, AC: anal cancer, pDC: peptide-pulsed dendritic cell, S.tumors: solid tumors, VV: viral vector-based vaccine, mPM: malignant pleural mesothelioma.

**Table 2:** Parameters used in the correlative studies.

Parameters	Predicted Percentage of Subjects in the MP:			Analysis Performed
	Number of Vaccine-Specific Epitopes	Number of Vaccine-Specific Proteins	Threshold of HLA Alleles Binding the Epitope (*n*)	
In Silico IRR (*n* × HLA)	≥ 1	≥ 1	≥ 1, ≥ 2, ≥ 3 or ≥ 4	Measured and predicted IRR correlations
In Silico multi-epitope IRR (*n* × HLA)	≥ 2	≥ 1	≥ 1, ≥ 2, ≥ 3 or ≥ 4	Measured and predicted CRR correlations
In Silico multi-Ag IRR (*n* × HLA)	≥ 2	≥ 2	≥ 1, ≥ 2, ≥ 3 or ≥ 4	Measured and predicted CRR correlations

## Data Availability

The data that support the findings of this study are available on request from the corresponding author.

## Supplementary Materials

The following are available online at https://www.mdpi.com/article/10.3390/cells10113048/s1, Table S1. Coverage of the HLA alleles in the MP, Table S2. Receiver Operating Characteristic (ROC) curve AUC values of the *in silico* IRR predictions, Figure S1. Pairwise Chi square analysis of measured and predicted IRRs, grouped by vaccine types, Figure S2. Pairwise Chi square analysis of measured CRRs and predicted multi-epitope IRRs, grouped by vaccine types.

## Appendix

The following tables are in an appendix excel file:

**Table A1:** Immune- and clinical response rates reported in the referenced studies. When the same vaccine was used in multiple trials the response rates were combined.

Vaccine	IRR	CRR	Combined IRR	Combined CRR	Ref.	Included in Analysis (X)					
						IRR-CRR Correlations	IRR in CTs with/without HLA Preselection	High Binding, Low Binding Epitope Vaccines	Measured and *In Silico* IRR Correlations	Measured CRR-*In Silico* IRR Correlations	MultiAg Correlations
**9-peptide breast cancer vaccine**	36%	---	36%	---	[37]		X		X		
**AFP-derived peptides**	33%	7%	33%	7%	[38]	X	X		X	X	
**Antigen-pulsed DC vaccine**	38%	---	38%	---	[39]		X		X		
**CD34 DC vaccine**	44%	---	44%	---	[40]		X		X		
**CV9103 mRNA vaccine**	80%	---	80%	---	[41,42]		X		X		
**DCCP peptide vaccine**	56%	11%	56%	11%	[23]	X	X		X	X	X
**DPX0907 peptide vaccine**	61%	---	61%	---	[43,44]		X		X		
**Elenagen pDNA vaccine**	---	0%	---	0%	[45]					X	
**EMD640744 peptide vaccine**	63%	---	63%	---	[22]		X		X		
**Five-peptide cancer vaccine**	---	11%	---	11%	[46]					X	X
**GAA peptides vaccine**	100%	29%	71%	20%	[47]	X	X	X	X	X	X
	55%	15%			[48]						
**GL-0817 (MAGE-A3 Trojan)**	0%	0%	33%	0%	[49]	X	X		X	X	
	33%	0%			[50]		X				
	57%	---			[51]		X				
**Glypican-3 peptide vaccine (A24)**	33%	33%	64%	18%	[52,53]	X	X	X	X		
	100%	0%						X			
**Glypican-3 peptide vaccine (A02)**	67%	0%	53%	38%		X	X	X	X		
	45%	60%						X			
**GVX301 peptide vaccine**	64%	0%	64%	0%	[54]	X	X		X	X	
	
**HER1 vaccine**	42%		42%	---	[55]		X		X		
**HER2 B cell vaccine**			---	6%	[56]					X	
**Her2/neu peptide vaccine (p369)**	62%		62%	---	[57]		X		X		
**Her2/neu peptide vaccine (p688)**	31%			---				X	X		
**Her2/neu peptide vaccine (p971)**	54%			---				X	X		
**HIVIS (HIV gag)**	80%		80%	---	[58]		X		X		
**HIVIS (HIV RT)**	50%		50%	---	[58]		X		X		
**HPV-SLP**	83%	60%	87%	47%	[59]	X	X		X	X	X
	100%	60%			[60]		X				
	---	33%			[61]						
**ICT107**	33%		33%	---	[62]		X		X		
**IDO and survivin peptide vaccine**	58%	6%	58%	6%	[63]	X	X	X	X	X	X
**IDOlong peptide vaccine**	10%	10%	10%	10%	[64]	X	X		X	X	
**IMA901**	64%	---	67%	---	[5]		X		X		
	74%	---					X				
**IMA950**	63%	0%	81%	2%	[65]	X	X		X	X	X
	81%	3%			[66]		X				
	90%	---			[67]		X				
**ImMucin**	100%	47%	96%	47%	[68,69]	X	X		X	X	
**IMP321/LAG-3Ig + peptides vaccine**	40%	---	40%	---	[70]		X	X	X		
**INGN-225 p53 vaccine**	58%	4%	58%	4%	[71]	X	X		X	X	
**KIF20A-66 peptide vaccine**	70%	26%	70%	26%	[72]	X	X	X	X	X	
**KRM-20 vaccine**	40%	13%	40%	13%	[73]	X	X		X	X	X
**MART-1 Peptide Vaccine**	15%	---	15%	---	[74]		X	X	X		
**Melanoma peptide vaccine**	52%	12%	52%	12%	[75]	X	X		X	X	X
**MELITAC 12.1**	49%	---	49%	---	[24]		X		X		
**Multiepitope peptide vaccine**	89%	0%	89%	0%	[76]	X	X	X	X	X	X
**NY-ESO-1 OLP**	82%	---	82%	0%	[77]		X		X	X	
					[78]						
**NY-ESO-1f**	90%	0%	90%	0%	[79]	X	X		X	X	
**OCV-C02 vaccine**	63%	0%	63%	0%	[80]	X	X		X	X	X
**p53 SLP70-235**	21%	---	21%	---	[81]		X		X		
**p53 SLP70-248**	0%	---	69%		[82]	X	X				
	88%	0%		0%	[83]		X		X	X	
	100%	0%			[84]		X				
**p53MVA vaccine**	55%	9%	55%	9%	[85]	X	X		X	X	
**PepCan**	65%	52%	65%	52%	[86]	X	X		X	X	
**Peptide cocktail therapy**	---	11%	---	11%	[87]					X	X
	---	14%	---	14%						X	X
**Peptide vaccine 1**	54%	---	54%	---	[88]		X		X		
**Peptide vaccine 2**	---	3%	---	3%	[89]					X	X
**Peptide vaccine 3**	100%	25%	100%	25%	[90]	X	X		X	X	X
**pNGVL4a-CRT/E7(detox) vaccine**	---	30%	---	30%	[91]					X	
**PR1 Peptide Vaccine**	53%		53%	---	[21]		X	X	X		
**ProstVac**	45%	---	62%	---	[97]		X		X		
	50%	---			[93]		X				
	67%	---			[92]		X				
	67%	---			[94]		X				
	72%	---			[96]		X				
	76%	---			[95]		X				
**PSMA-Survivin pulsed autologous DC vaccine**	---	18%	---	18%	[98]					X	X
**PVX-410 peptide vaccine**	95%	5%	95%	5%	[99]	X	X		X	X	X
**RHAMM-R3**	44%	0%	44%	0%	[100]	X	X	X	X		
**S-288310 peptide vaccine**	67%	17%	67%	17%	[101]	X	X		X	X	X
**StimuVax**	21%		21%	---	[102]		X		X		
**SVN-2B peptide vaccine**	60%		60%	---	[103]		X	X	X		
**Synchrotope TA2M**	46%	---	46%	---	[104]				X		
**TARP vaccine**	80%		80%	---	[105]		X		X		
**TG4010**	---	0%	35%	3%	[106]	X	X		X	X	
	21%	---			[107]		X				
	26%	0%			[108]		X				
	38%	13%			[109]		X				
**TSPP peptide vaccine**	---	5%	---	16%	[110]					X	
	---	24%			[111]						
**VGX-3100**	78%	---	78%	50%	[112]	X	X		X	X	X
	---	50%			[113]						
Vx-001	51%	---	59%		[114]	X	X				
	58%	4%		4%	[115]		X		X	X	
	66%	7%			[117]		X				
	71%	0%			[116]		X				
**WT1 vaccine (1)**	64%	0%	59%	4%	[118]	X	X		X	X	
	40%	10%			[119]		X	X			
	86%	0%			[120]	X	X				
**WT1 peptide vaccine**	72%	6%	72%	6%	[121]	X	X	X	X		
**WT1 peptides**	100%	14%	100%	14%	[122]	X	X		X	X	
**WT1 vaccine (2)**	83%	---	83%	---	[123]		X		X		

**Table A2:** HLA alleles of the model population and their frequency according to the Catalog of common, intermediate and well-documented HLA alleles (CIWD) [129].

HLA Allele	Frequency in CIWD
HLA-A*02:01	0.24065
HLA-A*01:01	0.13343
HLA-C*07:01	0.11948
HLA-A*03:01	0.11852
HLA-C*07:02	0.11093
HLA-C*04:01	0.11062
HLA-B*07:02	0.10205
HLA-A*24:02	0.08948
HLA-C*06:02	0.08771
HLA-B*08:01	0.08462
HLA-B*44:02	0.06295
HLA-A*11:01	0.06098
HLA-C*03:04	0.06076
HLA-C*05:01	0.05597
HLA-C*12:03	0.05491
HLA-B*51:01	0.05432
HLA-B*35:01	0.05270
HLA-B*15:01	0.05000
HLA-B*18:01	0.04534
HLA-C*02:02	0.04281
HLA-B*44:03	0.04280
HLA-C*03:03	0.04237
HLA-B*40:01	0.03957
HLA-C*01:02	0.03512
HLA-A*26:01	0.03352
HLA-A*68:01	0.03338
HLA-A*32:01	0.03200
HLA-B*57:01	0.03134
HLA-B*27:05	0.02952
HLA-B*13:02	0.02899
HLA-C*15:02	0.02573
HLA-C*08:02	0.02483
HLA-A*31:01	0.02462
HLA-B*35:03	0.02344
HLA-C*16:01	0.02241
HLA-B*38:01	0.02218
HLA-A*29:02	0.02166
HLA-A*25:01	0.02124
HLA-A*23:01	0.02096
HLA-B*14:02	0.02042
HLA-C*07:04	0.01709
HLA-B*52:01	0.01659
HLA-A*30:01	0.01589
HLA-B*40:02	0.01534
HLA-C*12:02	0.01526
HLA-B*49:01	0.01498
HLA-B*55:01	0.01438
HLA-C*14:02	0.01383
HLA-A*33:03	0.01360
HLA-B*37:01	0.01334
HLA-C*17:01	0.01273
HLA-B*58:01	0.01224
HLA-B*50:01	0.01189
HLA-B*39:01	0.01158
HLA-B*35:02	0.01027
HLA-A*02:05	0.00878
HLA-A*30:02	0.00838
HLA-A*68:02	0.00834
HLA-B*56:01	0.00784
HLA-C*03:02	0.00779
HLA-A*33:01	0.00749
HLA-B*40:06	0.00719
HLA-B*53:01	0.00696
HLA-B*45:01	0.00604
HLA-B*07:05	0.00593
HLA-B*41:01	0.00592
HLA-B*41:02	0.00579
HLA-B*14:01	0.00578
HLA-A*02:06	0.00569
HLA-C*15:05	0.00537
HLA-C*08:01	0.00515
HLA-B*39:06	0.00514
HLA-A*02:11	0.00463
HLA-A*66:01	0.00463
HLA-A*29:01	0.00413
HLA-A*03:02	0.00368
HLA-B*15:03	0.00337
HLA-B*15:18	0.00281
HLA-C*02:10	0.00275
HLA-B*15:02	0.00261
HLA-A*02:02	0.00239
HLA-A*24:03	0.00236
HLA-B*48:01	0.00229
HLA-B*13:01	0.00219
HLA-B*46:01	0.00215
HLA-C*16:04	0.00208
HLA-A*74:01	0.00200
HLA-B*57:03	0.00193
HLA-B*42:01	0.00193
HLA-A*02:07	0.00183
HLA-A*30:04	0.00183
HLA-A*02:03	0.00174
HLA-B*38:02	0.00171
HLA-A*34:02	0.00162
HLA-B*39:05	0.00159
HLA-B*15:10	0.00145
HLA-C*18:01	0.00136
HLA-B*58:02	0.00136
HLA-A*36:01	0.00097
HLA-B*15:16	0.00095
HLA-A*68:03	0.00084
HLA-B*54:01	0.00075
HLA-C*08:03	0.00073
HLA-B*35:17	0.00073
HLA-B*81:01	0.00067
HLA-C*14:03	0.00067
HLA-A*11:02	0.00056
HLA-B*39:24	0.00052
HLA-B*15:07	0.00049
HLA-A*80:01	0.00045
HLA-B*57:02	0.00045
HLA-B*35:43	0.00044
HLA-B*39:10	0.00043
HLA-B*42:02	0.00041
HLA-B*27:04	0.00041
HLA-C*08:04	0.00036
HLA-B*78:01	0.00033
HLA-C*12:04	0.00030
HLA-B*39:09	0.00029
HLA-A*66:02	0.00028
HLA-A*02:22	0.00025
HLA-B*15:04	0.00021
HLA-B*40:04	0.00021
HLA-B*15:11	0.00020
HLA-B*67:01	0.00014
HLA-B*35:20	0.00013
HLA-A*11:03	0.00013
HLA-C*04:04	0.00012
HLA-A*29:10	0.00012
HLA-B*14:03	0.00010
HLA-A*26:02	0.00009
HLA-B*59:01	0.00009
HLA-C*01:03	0.00008
HLA-B*40:11	0.00007
HLA-A*66:03	0.00007
HLA-A*26:03	0.00006
HLA-A*02:24	0.00006
HLA-B*39:13	0.00004
HLA-C*06:06	0.00003
HLA-A*02:14	0.00002
HLA-A*43:01	0.00002
HLA-A*36:03	0.00001
HLA-B*40:64	0.00001
HLA-A*02:87	0.00000
HLA-C*05:11	0.00000
HLA-A*03:17	0.00000
HLA-A*24:04	0.00000
HLA-B*13:03	0.00000
HLA-C*06:11	0.00000
HLA-A*24:24	0.00000
HLA-B*41:04	0.00000
HLA-B*49:02	0.00000
