# Supplementary material for: In Silico Model Estimates the Clinical Trial Outcome of Cancer Vaccines
Source: Cells. 2021 Nov 5;10(11):3048. doi: 10.3390/cells10113048 (PMC8616443; doi:10.3390/cells10113048)
Supplement: Supplementary file 1 [file cells-10-03048-s001.zip › cells-1421537-supplementary.pdf]

Table S1. Coverage of the HLA alleles in the MP. Calculations are based on the CIWD [129] frequencies. The 4818 HLA class I alleles included in the CIWD is considered as 1.00.

Restriction of the Subpopulation	Sample Size in MP (n)	CIWD Coverage (n = 8,077,802)
**Model population**	433	0.974
**non A24:02**	359	0.936
**A01, A02, A03, A24, or B07**	324	0.970
**A01, A02, or A03**	262	0.964
**A02 or A24**	240	0.963
**A01 or A02**	225	0.960
**A02:01 or A24:02**	200	0.949
**A02**	180	0.951
**A02:01**	134	0.936
**A24**	78	0.902
**A24:02**	74	0.894

Table S2. Receiver Operating Characteristic (ROC) curve AUC values of the *in silico* IRR predictions. Success threshold of the measured IRR varied between 30% and 80% (analyzed dataset: 59 data pairs covering 79 CTs with 55 vaccines).

IRR Threshold	ROC Curve AUC	Total Points Analyzed/Points Above IRR Threshold
30%	0.79	59/55
40%	0.66	59/48
50%	0.63	59/41
60%	0.71	59/29
70%	0.75	59/17
80%	0.71	59/13

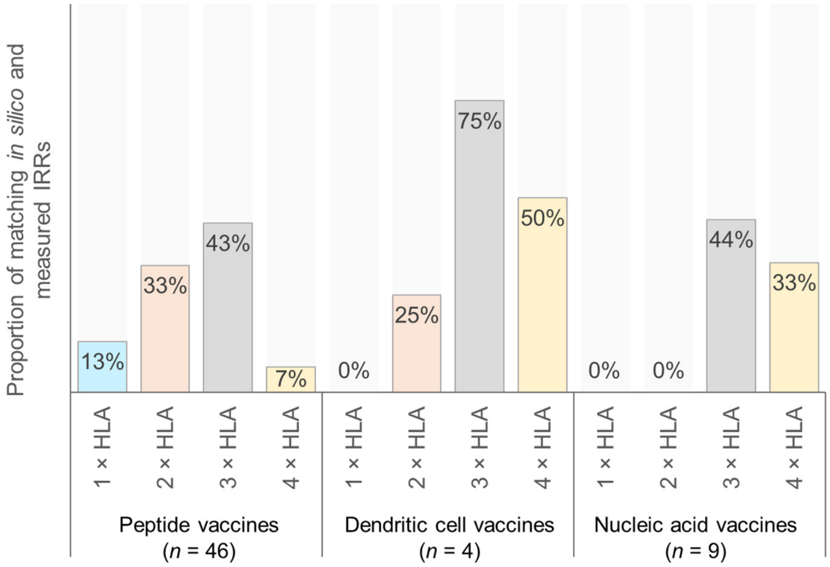

Figure S1. Pairwise Chi square analysis of measured and predicted IRRs, grouped by vaccine types. Peptide vaccines (46 data pairs), dendritic cell vaccines (4 data pairs), nucleic acid vaccines covering plasmid DNA, viral vector-based, and mRNA vaccines (9 data pairs). Bars represent the proportion of the analyzed data pairs where difference was not significant ($p > 0.05$). Analyzed dataset was the same as used for Figure 3 with the following

difference: in one CT [75] the vaccine was administered as different types for 2 arms: one received the vaccine as peptides and one as dendritic cells pre-pulsed with the same peptides. The CT results were described separately, thus for this analysis both results were included as two separate data pairs. Another CT [105] with similar settings was left out from this analysis compared to data in Figure 3), because there the results were only described for the whole study population and not for the separate arms. In total the figure shows analysis of 59 data pairs covering 78 CTs with 54 vaccines.

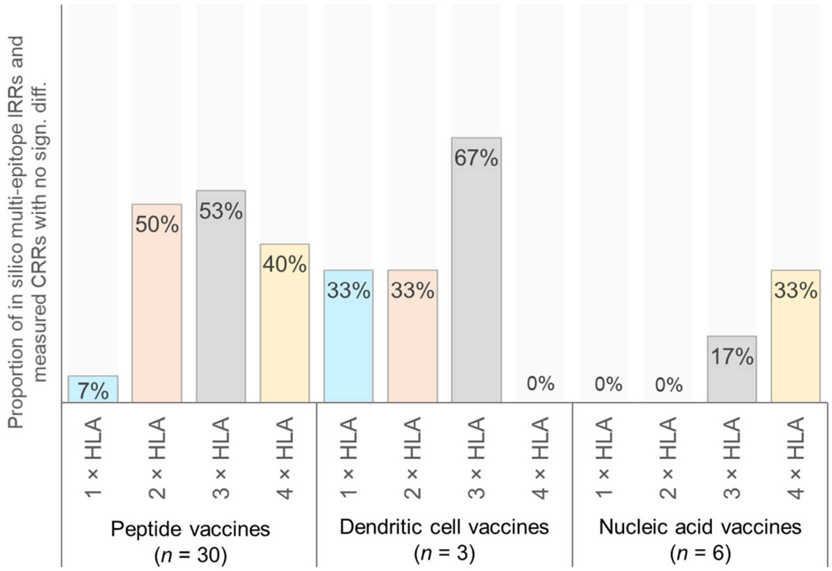

Figure S2. Pairwise Chi square analysis of measured CRRs and predicted multi-epitope IRRs, grouped by vaccine types. Peptide vaccines (30 data pairs), dendritic cell vaccines (3 data pairs), nucleic acid vaccines covering plasmid DNA and viral vector-based vaccines (6 data pairs); bars represent the proportion of the analyzed data pairs where difference was not significant ($p > 0.05$). Note: Analyzed dataset was the same as used for Figure 5 (49 CTs with 31 vaccines), with the following difference: in one CT [75] the vaccine was administered as different types for 2 arms: one received the vaccine as peptides and one as dendritic cells pre-pulsed with the same peptides. The clinical results were described separately, thus for this analysis both results were included as two separate data pairs.
